# Sleep-Disordered Breathing Is Associated with Metabolic Syndrome in Outpatients with Diabetes Mellitus Type 2

**DOI:** 10.1155/2019/8417575

**Published:** 2019-04-18

**Authors:** K. Neumann, M. Arzt, I. Heid, C. Böger, S. Stadler

**Affiliations:** ^1^Department of Internal Medicine II, University Hospital Regensburg, Regensburg, Germany; ^2^Department of Genetic Epidemiology, University of Regensburg, Regensburg, Germany; ^3^Department of Nephrology, University Hospital Regensburg, Regensburg, Germany

## Abstract

**Background:**

Metabolic syndrome (MS) and sleep-disordered breathing (SDB) are highly prevalent in patients with diabetes mellitus type 2 (DM2). The present study examined whether there is an independent association between SDB and MS in a sample of outpatients with DM2.

**Methods:**

MS was determined in 679 patients of the DIACORE-SDB substudy, a study of outpatients with DM2. According to the National Cholesterol Education Program (NCEP) criteria, MS is defined by at least three of the following five criteria: waist circumference of >102 cm (men)/>88 cm (women), blood pressure of ≥130/85 mmHg, a fasting triglyceride level of >150 mg/dl, high-density lipoprotein (HDL) of <40 mg/dl (men)/<50 mg/dl (women), and a fasting glucose level of ≥110 mg/dl. The apnea-hypopnea index (AHI) was assessed with a 2-channel ambulatory monitoring device and used to define the severity of SDB (AHI < 15.0: no/mild SDB; AHI 15.0-29.9: moderate SDB; AHI ≥ 30.0: severe SDB).

**Results:**

228 (34%) of the 679 participants (mean age 66 years, mean body mass index (BMI) 31.2 kg/m^2^, and mean AHI 14/hour) had SDB. MS was significantly more frequent in patients with more severe SDB (no/mild SDB vs. moderate SDB vs. severe SDB: 72% vs. 79% vs. 85%, respectively, *p* = 0.038). Logistic regression analysis adjusted for sex, age, obesity (BMI ≥ 30 kg/m^2^), and the HOMA index showed a significant association between the AHI and the presence of MS (OR (95%CI) = 1.039 (1.011; 1.068); *p* = 0.007). Further, male sex, obesity, and the HOMA index were significantly associated with MS.

**Conclusion:**

SDB is significantly and independently associated with MS in outpatients with DM2.

## 1. Introduction

The most common type of sleep-disordered breathing (SDB) is obstructive sleep apnea (OSA) [1]. OSA is characterised by recurrent collapse of the upper airway during sleep leading to oxygen desaturation with consecutive arousals from sleep [2, 3]. The pattern of desaturation and reoxygenation results in intermittent hypoxia, which is the main reason for metabolic dysfunction in SDB and is associated with the components of metabolic syndrome, which means hypertension, visceral obesity, pathological glucose tolerance, and dyslipidaemia [4–7]. The pathological mechanisms of SDB that cause hypertension include baroreflex impairment as well as hypoxia-induced activation of chemoreflex sensors, which increase both the sympathetic tone and peripheral vascular resistance [8–10]. Endothelial dysfunction due to hypoxia and oxidative stress also contribute to cardiovascular disease and hypertension [11]. Obesity is strongly associated with OSA in a bidirectional manner: visceral obesity is a risk factor of OSA [12–14]. The accumulation of visceral fat reduces lung volume and thoracic compliance, thus generating negative thoracic pressure, which subsequently leads to pharyngeal occlusion [14]. In addition, the deposition of adipose tissue in the upper airway facilitates collapsibility by narrowing pharyngeal patency [15]. At the same time, OSA leads to weight gain mostly due to endocrine dysregulation and physical inactivity because of daytime sleepiness [13, 16–18]. Endocrine alterations and fragmentation of sleep are also involved in impaired glucose metabolism, leading to pathological glucose tolerance and insulin resistance [19–21]. Thus, SDB increases the risk of developing DM2 [1, 22]. The association between SDB and dyslipidaemia, defined as an increase in triglyceride and a decrease in HDL levels, also contributes to intermittent hypoxia [23]. Treatment of SDB includes weight loss as well as therapy with continuous positive airway pressure (CPAP), which lowers blood pressure and improves glucose metabolism as well as the lipid profile [1, 10, 13, 22, 24, 25].

Overall, MS is strongly associated with SDB, and the reported prevalence ranges from 23% to 87% [5]. The present study examined whether there is an independent association between SDB and MS in a sample of outpatients with DM2.

## 2. Material and Methods

### 2.1. Study Design

The examined patients were participants of the DIACORE- (DIAbetes COhoRtE-) SDB substudy, a prospectively designed study of patients with DM2. Major diabetologists and medical insurance companies invited outpatients with DM2 in written form to participate in the study. Patients previously treated at the Department of Internal Medicine of the University Hospital Regensburg were also invited [26]. The diabetic status was determined by assessing diabetes medication or by validating self-report. Patients underwent a standardized physical examination and biosampling and had to fill in an online questionnaire [26]. Of 1036 individuals invited to participate in the DIACORE-SDB substudy, 721 agreed and were tested with a two-channel respiratory monitor (ApneaLink®, ResMed) [27]. Complete SDB parameters were recorded for 679 patients (94% of the 721 tested). MS could not be determined in two patients because of missing data on waist circumference and the triglyceride level. Thus, 677 participants were analysed with regard to the presence of MS. Follow-up is currently ongoing, so that the cross-sectional baseline data was used for the present investigation.

The protocol, the data protection strategy, and the study procedures were approved by the Ethics Committees of the participating institutes and were in accordance with the Declaration of Helsinki. Patients participated in the DIACORE study only after providing informed written consent.

### 2.2. Study Population

All DM2 outpatients living in the city and district of Regensburg were eligible for participating in the DIACORE-SDB substudy. Further inclusion criteria were the ability to fully understand the study information, to provide written informed consent, age ≥ 18 years, and self-reported Caucasian ethnicity [26]. Exclusion criteria were chronic renal replacement therapy (haemodialysis, peritoneal dialysis, or transplantation), history of active malignancy within the past five years, presence of an autoimmune disease potentially affecting kidney function, haemochromatosis, known pancreoprivic or self-reported type 1 diabetes mellitus, acute infection, fever, pregnancy, chronic viral hepatitis, and HIV infection [26]. Patients were included in the DIACORE-SDB substudy if they consented to undergo SDB screening and excluded if they currently used positive airway pressure therapy [27].

### 2.3. Assessment of SDB

SDB was assessed with the portable ApneaLink device (ResMed, Sydney, Australia) consisting of a nasal cannula and an oxygen clip to measure nasal flow and pulse oximetry. Trained study personnel instructed the participants on how to use the device at home. Several studies have validated the ApneaLink device (ResMed, Sydney, Australia) for the screening of SDB [28, 29]. The AHI, oxygen desaturation index, mean oxygen saturation, and minimum SpO_2_ were assessed. The default settings of the screening device were used for the definitions of apnea, hypopnea, and desaturation: apnea was defined as a ≥80% decrease in airflow for ≥10 seconds, hypopnea as a decrease in airflow by ≥50-80% versus baseline for ≥10 seconds, and desaturation as a ≥4% decrease in oxygen saturation [27]. The cut-off for the diagnosis of SDB was an AHI ≥ 15/h. Patients with an AHI < 15/h were assumed to have no or mild SDB. An AHI ≥ 15 up to 29 was defined as moderate SDB and an AHI ≥ 30 as severe SDB [24]. A differentiation between obstructive and central sleep apnea was not possible because of the absence of a breast belt. Daytime sleepiness was assessed by means of the Epworth Sleepiness Scale (ESS), and a score of ≥11 was considered as excessive daytime sleepiness [30].

### 2.4. Assessment of Metabolic Syndrome

According to the NCEP criteria, MS is defined by at least three of the following five criteria [4]: visceral obesity, defined by a waist circumference of >102 cm in men or > 88 cm in women; dyslipidaemia, defined by high-density lipoprotein (HDL) of <40 mg/dl in men or < 50 mg/dl in women; a fasting triglyceride level of >150 mg/dl or use of triglyceride-lowering medication; hypertension, defined by blood pressure of ≥130/85 mmHg or use of antihypertensive medication; and presence of a pathological glucose tolerance with a fasting glucose level of ≥110 mg/dl.

Weight in light clothing was measured with a digital scale. Blood pressure and heart rate were measured with a vital signs monitor after the patient had been sitting at rest for at least five minutes. Waist circumference is defined as the smallest circumference between the upper iliac crest and the lower coastal margin. In case of obesity, waist circumference was measured midway between the upper iliac crest and lower costal margin. Blood samples (serum gel, EDTA, and sodium fluoride (Sarstedt, Germany) and PAXgene tubes (PreAnalytix GmbH, Switzerland)) were taken after the patient had been sitting at rest for 15 minutes [26].

### 2.5. Statistical Analysis

Descriptive data are presented as the mean (±SD). Normally distributed values of baseline characteristics were evaluated with Student's unpaired two-sided *t*-test. Metabolic parameters were compared with increasing severity of SDB (no/mild, moderate, and severe) by one factorial variance analysis (ANOVA) and the post hoc test (Bonferroni). The influence of the AHI on the presence of the metabolic syndrome and its criteria were assessed with logistic regression models. Known modulators such as age, sex, obesity, and insulin resistance were included as covariates. Insulin resistance was assessed by means of the Homeostasis Model Assessment index (HOMA index) that has been validated in previous studies [31–33]. Obesity was defined as a body mass index (BMI) of ≥30 kg/m^2^. Results are given as the odds ratio and 95% confidence interval; *p* values of <0.05 were considered significant. Data were analysed with the SPSS statistical software package (SPSS 23.0, IBM SPSS Statistics, Armonk, New York, USA).

## 3. Results

### 3.1. Patient Characteristics

**Figure 1:**
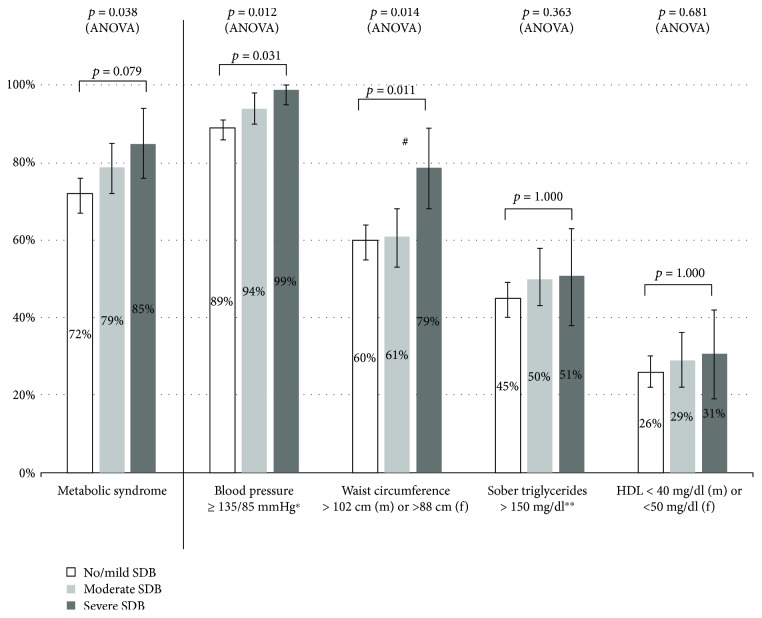
Prevalence of NCEP criteria (except elevated fasting glucose) in participants with no or mild, moderate, and severe SDB. Comparison of the prevalence of NCEP criteria with ANOVA and the post hoc test (Bonferroni) between no/mild and severe SDB. Results are shown with 95% confidence intervals. m: male, f: female; ^∗^or on antihypertensive medication, ^∗∗^or on triglyceride-lowering medication. ^#^Significant difference between moderate and severe SDB (*p* = 0.041).

The 679 patients of the SDB substudy (Figure 1) had a mean age of 65.6 years, and 61% were men. Patients were mostly obese (mean BMI 31.2 kg/m^2^), and the mean duration of DM2 was 10.2 years. Anamnesis of medication showed that 81% of participants received antihypertensive medication, 47% cholesterol-lowering agents, and 85% antidiabetic agents. 27% of patients required insulin therapy (Table 1).

### 3.2. Characteristics according to Severity of SDB

Patients were classified into three groups according to the severity of SDB. Of the 228 patients with SDB, 163 had moderate (AHI ≥ 15/h and <30/h) and 65 severe (AHI ≥ 30/h) SDB. Baseline characteristics were compared among the three groups. Patients with SDB were predominantly older, male, and mostly obese with a significantly higher waist circumference, higher waist-hip ratio, and higher systolic blood pressure as well as a lower HDL level (Table 2).

### 3.3. SDB and MS

According to the NCEP criteria, MS was prevalent in 75% of the participants, and 80% of the patients with SDB had MS. The comparison of the severity of SDB among the three groups showed that MS as well as its components visceral obesity and hypertension was significantly more frequent in patients with more severe SDB (Figure 1). The criterion of elevated fasting glucose level was excluded, because DM2 was prevalent in all participants.

After adjusting for sex, age (in decades), obesity (defined as BMI ≥ 30 kg/m^2^), and the HOMA index in a multivariate regression analysis, the AHI was significantly and independently associated with the presence of MS (OR (95% CI) = 1.039 (1.011; 1.068); *p* = 0.007). Male sex, obesity, and the HOMA index were independent modulators of MS. In the same multivariable regression, the AHI was also significantly associated with several components of MS: elevated waist circumference (OR (95% CI) = 1.031 (1.006; 1.056); *p* = 0.014), hypertension (OR (95% CI) = 1.049 (1.000; 1.100); *p* = 0.048), and hypertriglyceridemia (OR (95% CI) = 1.018 (1.002; 1.035); *p* = 0.029) (Table 3).

## 4. Discussion

The present study shows that, in patients with DM2, MS and its criteria hypertension and visceral obesity were significantly more frequent with increasing severity of SDB. Logistic regression analysis yielded a significant and independent association between increasing AHI and the prevalence of MS as well as visceral obesity, hypertension, and hypertriglyceridemia.

Our data confirm the results of previous studies describing a higher prevalence of MS in patients with SDB [34–38] (Table 4). However, the present study complements previous studies in the following manner.

First, previous studies were not conducted in a sample of outpatients with DM2. Although some studies included patients with DM2 or with pathological glucose tolerance, the percentage is still rather low (5-30%) [5, 34, 37–40]. Thus, to our knowledge, the present study is the first to exclusively analyse the association of SDB with MS and its components in patients with DM2.

Second, several previous studies defined SDB as an AHI ≥ 5 or ≥10 [5, 34, 37–41]. However, our participants with an AHI < 15/h were not sleepy, which was shown by the low ESS (Table 2); thus, they did not require any treatment. For this reason, we defined clinically relevant SDB as an AHI ≥ 15 and used the recommended classification of SDB severity [24].

With respect to the prevalence of MS criteria, our findings are mostly consistent with previous study results. The association of SDB with hypertension [34, 37–40] and obesity [37, 38], as in the present study, is well known. Nevertheless, Kono et al. and Lin et al. found a significant association of SDB with the components of MS in nonobese patients [39, 40]. Parish et al. did not find any significant differences in the BMI between patients with and without OSA and assessed hypertension as the main factor for MS in patients with OSA [34]. Although most of our patients were obese and obesity remains to be significantly associated with MS, we found an independent association of the AHI with MS in a logistic regression model.

Results concerning an association between SDB and dyslipidaemia according to NCEP criteria have been inconsistent [34, 37, 39]. In the present study, there was no significant association between SDB and dyslipidaemia. However, a significant association between the severity of SDB and decreased HDL could be found. Also, other studies which were calculated with continuous variables of dyslipidaemia instead of NCEP criteria showed an association between SDB and the lipid profile, such as elevated triglycerides and low HDL [35–39, 42–44].

SDB is associated with pathological glucose tolerance and insulin resistance [19, 20]. In the present study, the duration of DM2 was significantly longer in patients with SDB than in patients with no or mild SDB (11.4 ± 9.0 vs. 9.5 ± 7.4 years; *p* = 0.005).

With respect to the association of SDB and components of MS, Coughlin et al. discussed in their review whether SDB may be a component of MS [45]. When examining patients of the Wisconsin Sleep Cohort Study for metabolic parameters, Nieto et al. [41]. detected a significant association of SDB with MS independent of sex, age, BMI, sympathetic, and neuroendocrine parameters; thus, the authors considered SDB (defined as an AHI ≥ 5) to be a component of MS [41]. The present study also showed an increasing risk of MS as well as of visceral obesity, hypertension, and hypertriglyceridemia with a rising AHI. However, using a high cut-off and defining SDB as an AHI ≥ 15, the prevalence of SDB in patients with MS was rather low (36%) compared to the prevalence of hypertension (97%), visceral obesity (80%), and hypertriglyceridemia (61%) in patients with MS; therefore, there is no sufficient evidence to substantiate the claim that SDB is an integral component of MS.

The strength of our study is its large sample size with central data management and standardized protocols [26]. Furthermore, to our knowledge, this is the first study examining the association between SDB and MS in outpatients with DM2. There are some limitations that warrant discussion: First, a distinction between central and obstructive sleep apnea was not possible because of the use of a portable yet validated and established [28, 29] SDB monitoring device instead of polysomnography. Second, as our data stem from a cross-sectional analysis, we were only able to assess an association between SDB and MS but could not prove any causality. Third, since 100% of participants of the DIACORE-SDB substudy have diabetes, results cannot be extrapolated to patients with milder forms of altered glucose metabolism (fasting glucose < 126 mg/dl).

In summary, our findings showed that SDB is significantly and independently associated with MS in outpatients with DM2. As previous randomized controlled trials of CPAP treatment in patients with DM2 fell short of identifying an effect on glucose metabolism [46], future large-scaled long-term interventional studies are required.

## Figures and Tables

**Table 1:** Baseline characteristics.

	*N* = 679
Age (years)	65.6 ± 8.8
Sex, male (n (%))	412 (60.7%)
AHI/h	14 ± 13
*Metabolic parameters*	
Waist circumference (cm)	100.8 ± 16.6
BMI (kg/m^2^)	31.2 ± 5.5
Systolic BP (mmHg)	138 ± 18
Diastolic BP (mmHg)	75 ± 10
HDL (mg/dl)	52.8 ± 14.9
Triglycerides (mg/dl)	171.4 ± 131.5
Duration of DM2 (years)	10.2 ± 8.0
HbA1c (%)	6.8 ± 1.1
(mmol/mol)	51 ± 12
HOMA index^∗^	6.2 ± 7.5
*Medication N (%)*	
Antihypertensive medication	
Antihypertensive medication altogether	547 (81%)
ACE inhibitor	280 (41%)
Angiotensin receptor blocker	164 (24%)
Renin inhibitor	3 (<1%)
Calcium channel blocker	191 (28%)
Beta-blocker	322 (47%)
Diuretics	267 (39%)
Fat metabolism	
HMG-CoA-reductase inhibitor	318 (47%)
Diabetes medication	
Antidiabetic agents altogether	575 (85%)
Biguanide	459 (68%)
Incretin	153 (23%)
Sulfonylurea	127 (19%)
Glinide	26 (4%)
Alpha-glucosidase inhibitor	9 (1%)
Exenatide	6 (1%)
Insulin	181 (27%)

Results are provided as mean ± standard deviation or *n* (%). AHI: apnea-hypopnea index; BMI: body mass index; BP: blood pressure; HbA1c: haemoglobin A1c; HOMA: Homeostasis Model Assessment (fasting, use of long-acting insulin); DM2: diabetes mellitus type 2; HDL: high-density lipoprotein; ^∗^413 patients included.

**Table 2:** Characteristics according to severity of SDB.

	No/mild SDB (*N* = 451)	Moderate SDB (*N* = 163)	Severe SDB (*N* = 65)	*p* value	No/mild vs. moderate SDB	Moderate vs. severe SDB	No/mild vs. severe SDB
Mean AHI/h	7 ± 4	21 ± 4	46 ± 12	**<0.001**	**<0.001**	**<0.001**	**<0.001**
Waist circumference (cm)	98.8 ± 16.4	102.3 ± 16.1	111.5 ± 15.4	**<0.001**	0.054	**<0.001**	**<0.001**
Waist-hip ratio	0.95 ± 0.08	0.98 ± 0.07	1.00 ± 0.08	**<0.001**	**<0.001**	0.093	**<0.001**
BMI (kg/m^2^)	30.4 ± 5.1	31.3 ± 5.2	34.0 ± 6.8	**<0.001**	0.139	**0.002**	**<0.001**
Systolic BP (mmHg)	137 ± 18	140 ± 19	141 ± 17	**0.041**	0.135	1.00	0.179
Diastolic BP (mmHg)	74 ± 10	75 ± 10	76 ± 10	0.393	0.980	1.00	0.794
HDL (mg/dl)	54.0 ± 15.5	51.0 ± 13.6	49.6 ± 12.9	**0.016**	0.080	1.00	0.080
Triglycerides (mg/dl)	165.7 ± 125.1	181.1 ± 155.3	186.5 ± 107.0	0.273	0.597	1.00	0.699
HbA1c (%)	6.8 ± 1.1	6.9 ± 1.2	6.6 ± 0.7	0.111	0.867	0.110	0.340
(mmol/mol)	51 ± 12	52 ± 13	49 ± 7				
HOMA index^∗^	5.6 ± 5.9	6.7 ± 9.1	7.9 ± 10.9	0.137	0.607	1.00	0.242
ESS	5 ± 3	5 ± 4	6 ± 3	0.148	1.00	0.863	0.195
Number of NCEP criteria	3 ± 1	3 ± 1	4 ± 1	**0.009**	0.306	0.350	**0.013**

Results are provided as mean ± standard deviation and *p* value (ANOVA). *p* values among groups were assessed by post hoc test (Bonferroni). AHI: apnea-hypopnea index; BMI: body mass index; BP: blood pressure; HDL: high-density lipoprotein; HbA1c: haemoglobin A1c; HOMA: Homeostasis Model Assessment (fasting, use of long-acting insulin), ESS: Epworth Sleepiness Scale; ^∗^413 patients included.

**Table 3:** Multivariable logistic regression analysis.

	Metabolic syndrome		Waist circumference > 102 cm (m) or >88 (f)		BP ≥ 130/85 mmHg^∗^		Triglycerides > 150 mg/dl^∗∗^		HDL <50 mg/dl (m) or <40 mg/dl (f)	
	OR (95% CI)	*p* value	OR (95% CI)	*p* value	OR (95% CI)	*p* value	OR (95% CI)	*p* value	OR (95% CI)	*p* value
AHI	1.039 (1.011; 1.068)	**0.007**	1.031 (1.006; 1.056)	**0.014**	1.049 (1.000; 1.100)	**0.048**	1.018 (1.002; 1.035)	**0.029**	1.012 (0.995; 1.030)	0.167
Sex	2.477 (1.377; 4.457)	**0.002**	6.874 (3.704; 12.757)	**<0.001**	0.620 (0.300; 1.284)	0.198	0.937 (0.611; 1.436)	0.765	1.372 (0.847; 2.222)	0.199
Age	1.010 (0.733; 1.392)	0.950	0.973 (0.689; 1. 373)	0.876	1.856 (1.253; 2.750)	**0.002**	0.909 (0.713; 1.161)	0.446	0.763 (0.578; 1.007)	0.056
Obesity	7.505 (3.689; 15.268)	**<0.001**	21.858 (11.129; 42.934)	**<0.001**	1.655 (0.733; 3.735)	0.225	1.411 (0.917; 2.172)	0.117	1.243 (0.760; 2.033)	0.386
HOMA index	1.313 (1.156; 1.491)	**<0.001**	1.106 (1.026; 1.192)	**0.008**	1.147 (0.996; 1.321)	0.058	1.038 (1.004; 1.074)	**0.029**	1.056 (1.021; 1.093)	**0.002**

Shown are adjusted odds ratios (OR) with 95% confidence intervals (95% CI) and *p* values. The variables used in the regression model were AHI, sex (male), age (decades), obesity (BMI ≥ 30), and the HOMA index. AHI: apnea-hypopnea index; HOMA: Homeostasis Model Assessment (fasting, use of long-acting insulin); ^∗^or on antihypertensive medication; ^∗∗^ or on triglyceride-lowering medication.

**Table 4:** Previous studies examining the association between SDB and MS.

	Study design and participants	Patients with pathological glucose tolerance	Definition and assessment of SDB	Main results
Neumann et al. (2019)	228 SDB patients, 451 controls; 61% men	100% (all participants had proven DM2)	SDB: AHI ≥ 15; ApneaLink	(i) In patients with DM2, MS as well as its criteria hypertension and visceral obesity was significantly more frequent in the case of more severe SDB

Bonsignore et al. [5]	529 OSA patients; 80% men	17% (DM2)	SDB: AHI ≥ 10; PSG	(i) The prevalence of MS increased with OSA severity (ii) Obesity and OSA led to metabolic abnormalities with different patterns between the two sexes (iii) Metabolic score increased with the HOMA index

Lin et al. [39]	113 OSA patients, 45 controls; 82% men; only nonobese subjects included; no difference in BMI among groups	18% (hyperglycaemia)	SDB: AHI ≥ 5; PSG	(i) Patients with OSA had significantly higher systolic blood pressure and triglyceride levels (ii) Dyslipidaemia, hypertension, and at least two of the NCEP criteria were significantly more frequent in the OSA group (iii) AHI was independently associated with increased triglycerides and insulin resistance (assessed with HOMA) in linear regression

Nieto et al. [41]	253 OSA patients, 293 controls; 56% men	Not given	Mild SDB: AHI 5-14.9, moderate to severe SDB: AHI ≥15 or CPAP PSG	(i) Logistic regression adjusted for age, sex, autonomic and neuroendocrine parameters, and BMI showed an association of MS with mild and moderate/severe SDB

Kono et al. [40]	42 OSA patients, 52 controls matched for age, BMI, and visceral fat accumulation; 100% men; only nonobese subjects included	5% (DM2)	SDB: AHI ≥ 5; PSG	(i) No significant differences in serum levels of triglycerides, HDL, and diastolic BP (ii) Prevalence of hyperglycaemia, dyslipidaemia, and hypertension was significantly higher in the OSA group (iii) Patients with OSA had more often at least two of the criteria hypertension, hyperglycaemia, and dyslipidaemia, independent of visceral fat obesity

Parish et al. [34]	146 OSA patients, 82 controls; 59% men	30% (hyperglycaemia)	SDB: AHI ≥ 5 and ≥10; PSG	(i) MS was more often present in patients with OSA (ii) Prevalence of hypertension was significantly higher in the OSA group (iii) No significant differences in hyperglycaemia and dyslipidaemia (iv) Prevalence of MS increased with severity of OSA

Gruber et al. [36]	38 OSA patients, 41 controls; percentage of men not given; MS is defined according to IDF	Not given	Minimal patient contact sleep diagnosis system (VISI-3, Stowood Scientific Instruments Ltd. (SSI), Oxford);	(i) The prevalence of MS was higher in the OSA group (ii) Logistic regression adjusted for age, BMI, and smoking showed an independent association of OSA and MS (iii) OSA was independently associated with the levels of triglycerides and glucose as well as the Epworth score values, whereas insulin resistance (assessed with HOMA) was not significant
Lam et al. [37]	95 OSA patients, 160 controls; 59% men	7% (DM2)	SDB: AHI ≥ 5; PSG	(i) Patients with OSA were five times more likely to have MS (ii) OSA was independently associated with MS and some of its components (iii) Prevalence of MS increased with OSA severity

Sasanabe et al. [38]	819 OSA patients, 89 controls; 86% men	22% (hyperglycaemia)	SDB: AHI ≥ 5; PSG	(i) MS was significantly more frequent in patients with OSA (ii) The risk of MS was associated with the severity of OSA (iii) Hypertension, dyslipidaemia, and visceral obesity were more common in patients with OSA

Coughlin et al. [35]	61 OSA patients, 43 controls; 100% men	Not given	SDB:AHI > 15; PSG	(i) Patients with OSA had a greater waist circumference and higher systolic and diastolic blood pressure, were more insulin-resistant (assessed with HOMA), and had lower HDL and a higher prevalence of MS (ii) Patients with OSA were 9.1 times more likely to have MS

DM2: diabetes mellitus type 2; AHI: apnea-hypopnea index; PSG: polysomnography; OSA: obstructive sleep apnea; MS: metabolic syndrome; BP: blood pressure; BMI: body mass index; IDF: International Diabetes Federation; HDL: high-density lipoprotein; HOMA: Homeostasis Model Assessment; CPAP: continuous positive airway pressure.

## Data Availability

The data of this study are available from the corresponding author upon request.
